# Post-viral lung diseases: the microbiota as a key player

**DOI:** 10.1183/23120541.00560-2024

**Published:** 2025-04-07

**Authors:** Sabine V. Stadler, Christophe von Garnier, Niki D. Ubags

**Affiliations:** Division of Pulmonary Medicine, Lausanne University Hospital (CHUV) and University of Lausanne, Lausanne, Switzerland

## Abstract

Viral infections of the respiratory tract can lead to chronic lung injury through immunopathological mechanisms that remain unclear. Communities of commensal bacteria colonising the respiratory tract, known as the respiratory tract microbiota, are altered in viral infections, which can contribute to inflammation, lung epithelial damage and subsequent development of lung disease. Emerging evidence on post-viral lung injury suggests an interplay between viral infections, immune responses and airway microbiota composition in the development of viral-induced lung diseases. In this review, we present the clinical characteristics of post-viral lung injury, along with the underlying immunopathological mechanisms and host–bacteria interactions, with a focus on influenza virus, respiratory syncytial virus and coronaviruses. Additionally, considering the important role of the airway microbiota in viral-induced pulmonary sequelae, we suggest key areas for future research on respiratory microbiota involvement in the development of post-viral lung diseases.

## Shareable abstract

**The airway microbiota acts as a fundamental cofactor in altered immune responses contributing to post-viral lung injury. Further investigation is needed to understand the interplay between the host and lung microenvironment after viral infection.** https://bit.ly/4eb6gii

## Introduction

Respiratory viral infections are among the most common diseases across all age groups, which can manifest as mild symptoms but also lead to severe disease [1]. Increasing evidence has revealed the occurrence of persisting lung injury after acute viral infections, accounting for high mortality and morbidity [2]. The recent coronavirus disease 2019 (COVID-19) is a major example, as pulmonary sequelae after severe acute respiratory syndrome (SARS)-CoV-2 infection have far-reaching consequences for patients, healthcare systems and the economy [3]. In fact, 18% of COVID-19 survivors are affected by persistent respiratory symptoms after acute disease, independently of disease severity [4]. Among respiratory viruses, influenza virus A and respiratory syncytial virus (RSV) have also been shown to induce post-acute lung injury [2, 4]. To date, the immunopathological mechanisms underlying post-viral lung diseases are not entirely understood, calling for the need to investigate the development of post-viral pulmonary sequelae.

The respiratory tract is colonised by commensal microbes (including bacteria, fungi and viruses), affecting lung health and disease [5]. The airway microbiota is known to regulate host immunity [6] and is involved in lung repair and disease progression [7]. Viral lung infection can disrupt the respiratory microbiota, leading to dysbiosis [8, 9]. The latter can, in turn, induce inflammation, tissue destruction and remodelling [7, 10, 11] and may ultimately affect pulmonary function [12]. Given the important role of the microbiota in respiratory diseases, it is essential to include our current knowledge on the microenvironment in research on post-viral lung injury.

**FIGURE 1:**
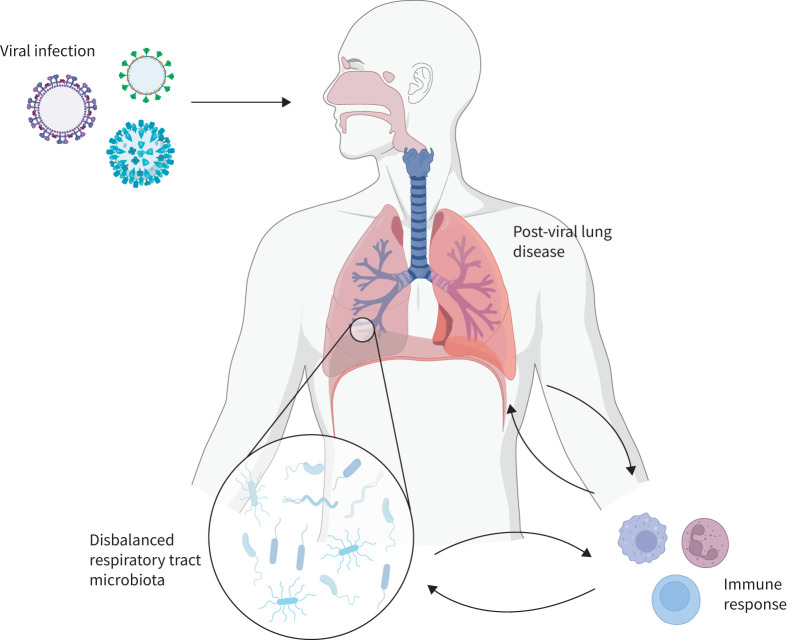
Host–bacteria interactions in post-viral lung diseases. Viral infections can lead to disruption of the respiratory tract microbiota. The latter plays a key role in the immunopathological mechanisms leading to post-viral lung diseases: disbalanced microbiota can lead to altered immune response, which in turn may affect bacterial community composition through the release of cytokines and interleukins. This vicious circle is further enhanced by the generated chronic inflammatory state and can lead to post-viral lung disease.

The respiratory tract microbiota might play a key role in the mechanisms leading to long-term lung injury after viral infections through host–bacteria interactions (figure 1). In this review, we describe the clinical characteristics of post-viral lung injury, the related immunopathological mechanisms, as well as post-acute airway microbiota profiles observed in three major respiratory viruses of concern (influenza virus, RSV and coronaviruses), which were selected given the paucity of literature in this field regarding other respiratory viruses. Finally, we highlight key areas for future research and provide future perspectives for research on respiratory microbiota in the development of post-viral lung diseases.

## Influenza

### Pulmonary sequelae after influenza virus infection

Influenza virus is one of the most prevalent viral pathogens associated with respiratory infection, with 5–15% of the global population being infected each year [13]. Until now most studies primarily focused on understanding acute pulmonary complications. Follow-up studies revealed the presence of pulmonary sequelae after influenza virus infection [14–16]. In previously hospitalised patients with H7N9 infection, chest computed tomography (CT) scans revealed pulmonary fibrosis and parenchymal opacifications persisting at 12 months after hospitalisation, along with diffusion capacity of the lung for carbon monoxide (*D*_LCO_) impairment in 77% of the patients, independently of the disease severity [14]. Although the subjects improved in lung function and radiological abnormalities in the first 6 months after the acute disease, these anomalies did not improve further at 24 months [14]. Similarly, in a 1-year follow-up study, 29% of patients with previously mild pandemic 2009 influenza A (H1N1) infection still had respiratory tract symptoms, and 55% suffered from abnormal lung function [15]. In more than 60% of patients with H1N1-induced acute respiratory distress syndrome (ARDS), a decreased *D*_LCO_ was reported at 1 year after the disease; however, ground-glass opacities (GGOs) were poorly prevalent at this time point [16]. Histological findings in the lungs of fatal influenza A cases showed that the lungs were defined by inflammation and oedema, as well as diffuse alveolar damage and fibrosis [17]. Despite the lack of additional follow-up studies, these findings emphasise the significance of chronic pulmonary sequelae after influenza virus infection and possible long-term consequences for patients.

### Mechanisms involved in post-influenza lung injury

To further characterise and understand the mechanisms involved in lung recovery and injury after influenza infection, experimental animal studies using influenza virus infection were used to investigate post-influenza lung sequelae. Adult mice infected with a nonlethal dose of mouse-adapted H1N1 (PR/8/34 strain) exhibited alveolitis and lung epithelial metaplasia, as well as increased expression of fibrotic genes (including chloride channel accessory 3 (Clca3), elastin and keratin 14) at 2 months post-infection [18]. This post-acute fibrotic state was confirmed in mice infected with influenza A virus (either WS/33 or PR8 strain), showing peribronchiolar and parenchymal fibrosis at 49 days post-infection. At that same time point, an increased production of interleukin (IL)-13 was linked to enhanced mucus production and airway hyperreactivity [19]. Similarly, in mice infected with Sendai virus, a parainfluenza virus, lung alveolar and interstitial CD68^+^ macrophages produced an elevated amount of IL-13 up to 1 month after infection [20]. The persistence of this inflammatory state might result from active viral RNA remnants remaining in the host's lung for several months, hence promoting immune cell stimulation persisting even after clearance of these viral antigens, thereby altering lung cell homeostasis [19]. Heaton *et al*. [21] demonstrated that murine lung epithelial cells, especially club cells surviving H1N1 infection, produced an increased amount of pro-inflammatory mediators (including CXCL10, CCL5 and CCL20), thereby inducing lung tissue damage. However, other cytokines, such as IL-22, have been shown to play an essential and protective role in lung epithelial repair after H1N1 infection [22, 23]. Specifically, IL-22 has a central function in maintaining epithelial cell integrity, reducing lung inflammation and enhancing antimicrobial activity of the lung epithelium [24–26].

### Respiratory tract microbiota after influenza virus infection

Cross-sectional studies using samples from humans and mice described differences in bacterial composition of the respiratory tract in the post-acute phase of influenza virus infection between infected and healthy subjects [27–29]. In the upper respiratory tract (URT), Gammaproteobacteria was the most prevalent taxa in patients infected with H1N1 during the acute and the post-acute state, whereas Actinobacteria dominated in the healthy group [27]. The subjects included in this study suffered from severe influenza infection with almost half of the subjects requiring hospitalisation and critical care. ARDS, which can occur following viral infection, has been shown to induce a translocation of gut bacteria to the lungs [28]. Therefore, a loss in the gut barrier integrity might, at least in part, explain the observed altered microbiota in the lungs after severe influenza infection. In line with these findings, experimental mice infected with H1N1/PR81 carried a more diverse lower respiratory tract (LRT) microbiota compared with noninfected controls, which increased rapidly in the acute phase and decreased during recovery [29]. The species diversity in infected mice did not restore to normal state at 4 weeks after infection. Moreover, a shift was observed in the dominant class, from Alphaproteobacteria to Gammaproteobacteria during the acute phase of influenza virus infection, as well as an increase in (facultative) anaerobe bacteria such as *Streptococcus* and *Staphylococcus* at 28 days post-infection [29]. Similarly, an intranasal challenge with live, attenuated influenza virus (FluMist, influenza vaccine) led to an increase in *Staphylococcus* present in the nasal cavity up to 6 weeks later [30]. Overall, these findings might be significant, as Gammaproteobacteria include anaerobe Gram-negative bacteria, which have the ability to metabolise inflammatory by-products to support their growth [19]. Gammaproteobacteria taxa include *Pseudomonas* *aeruginosa*, a bacteria associated with poor outcomes in chronic lung diseases, reiterating its possible role in the development of post-influenza lung injury [31–33].

**FIGURE 2:**
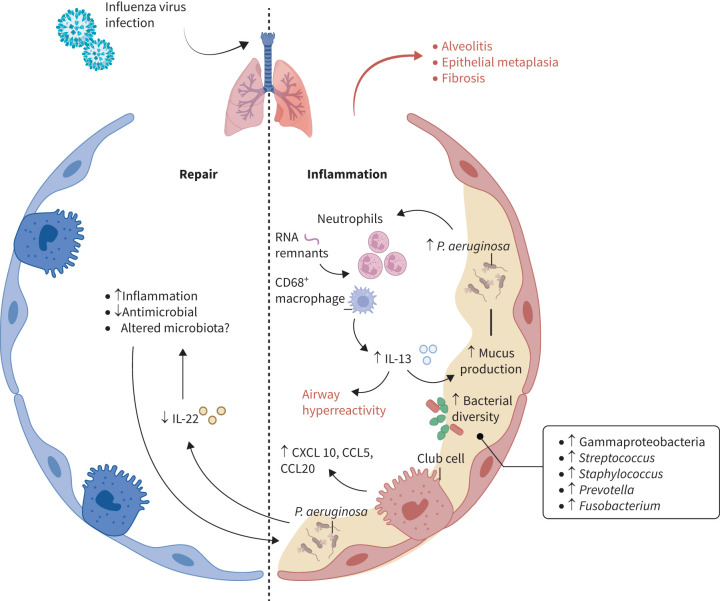
Host–bacterial interactions in post-influenza lung disease. In the post-acute influenza infection, lung alveolar and interstitial CD68^+^ macrophages produce an elevated amount of interleukin (IL)-13, enhancing mucus production and airway hyperreactivity [20]. This nutrient-rich environment provides advantageous conditions for *Pseudomonas* *aeruginosa* growth, perpetrating a vicious circle of chronic inflammation [34, 35]. Influenza RNA remnants might intensify this persistent inflammatory state [19]. *P.* *aeruginosa* promotes IL-22 degradation, protecting itself from the host's immunity and counteracting the protective role of IL-22 in lung epithelial repair after pandemic 2009 influenza A (H1N1) infection [37]. Club cells surviving acute H1N1 infection produce pro-inflammatory mediators such as CXCL10, CCL5 and CCL20, inducing lung tissue damage [21]. The observed post-acute inflammatory state is also characterised by an increased bacterial diversity, with a shift towards enhanced Gammaproteobacteria and increased *Streptococcus*, *Staphylococcus*, *Prevotella* and *Fusobacterium* [27, 29, 38].

Influenza infection can also induce chronic inflammation in the airways, through IL-13-mediated increase of airway hyperreactivity and mucus hypersecretion. Airway mucus provides a nutrient-rich environment, and along with an excessive settlement of neutrophils in the mucus, it provides anaerobic conditions that are advantageous for *P.* *aeruginosa* growth, outcompeting other members of the respiratory tract microbiome [34, 35]. This, in turn, triggers the immune system, perpetrating a vicious circle of chronic inflammation. *P. aeruginosa* might also regulate the IL-22 pathway. In fact, IL-22-deficient mice harbour an altered colonic microbiota, with an increase in Proteobacteria [36]. Although it remains to be clarified whether these effects on the host microbiota also occur in the pulmonary microenvironment, a study from Guillon *et al*. [37] showed that *P.* *aeruginosa* was able to degrade IL-22, thus protecting itself from the host's immunity. These studies indicate that the influenza-induced release of protective mediators such as IL-22 not only prevents aberrant lung recovery, but might also be of relevance in maintaining a balanced microbiota [37]. The respiratory tract microenvironment might therefore play a central role in the regulation of the IL-22 pathway involved in pulmonary repair after influenza virus infection (figure 2).

Nevertheless, there is conflicting evidence regarding the observed long-term alterations in airway microbiota, which may be, at least in part, related to viral load and symptom severity. In fact, two studies described a remarkable stability of the respiratory microbiota after influenza virus infection [38, 39]. Healthy volunteers challenged with intranasal influenza (H3N2) virus did not show a difference in bacterial richness in the URT compared with the control group, and the bacterial community composition was only slightly altered, showing an increase in *Prevotella* and *Fusobacterium* in patients with mild-to-moderate disease at 28 days following infection [38]. Moreover, in H5N1-infected mice, which exhibited only mild disease, the bacterial diversity in the LRT did not change over the course of infection [39]. Thus, infection with different influenza subtypes, varying sampling sites and sample collection methods, as well as the disease severity may partially explain these discrepancies.

Taken together, the influenza-mediated immune response might have a significant effect on the respiratory microbiota, disrupting the physiological balance and promoting the outgrowth of bacteria that benefit from the altered microenvironment. In turn, this dysbiosis promotes inflammation and abnormal lung repair, inducing post-viral pulmonary sequelae (figure 2).

## Respiratory syncytial virus

### Respiratory syncytial virus-induced lung injury

RSV represents a leading cause of upper and lower respiratory tract infections [40]. In children, over 60% of acute respiratory infections are caused by RSV [41]. Particularly, infants under 1 year of age are at high risk of developing severe and potentially fatal infection, known as bronchiolitis [41]. Over the last decades, significant long-term effects of RSV bronchiolitis on lung health have been described. Infants previously hospitalised with RSV bronchiolitis (at age <5 years) were at higher risk for the development of asthma and bronchial obstructive disease by the age of 7 years compared with the healthy control group, independent of family history of atopy [42, 43]. Similarly, among children having suffered from severe RSV infection during their first year of life, 48% were diagnosed with asthma by the age of 7 years [44]. On the other hand, children (under 3 years of age) with RSV infection that did not require hospitalisation had an increased risk for wheezing up to 6 years of age. This risk was found to decrease with age and reached nonsignificance at 13 years of age [45]. This indicates that severity of disease following RSV infection may differentially impact upon long-term lung health.

Newborns and young children are vulnerable for severe RSV infection and subsequent development of lung injury. After birth, the lung undergoes alveolarisation, meaning maturation and formation of the alveoli. This critical phase of lung development occurs mainly during the first 2 years of life, when the risk for severe RSV infection is the highest [46]. Alteration of this maturation process by severe inflammation might therefore generate extensive pulmonary damage. Even though the above studies underline altered lung health in childhood after RSV infection, adults with a history of viral bronchiolitis during infancy were also at higher risk for lung disease [47–49]. In fact, bronchial hyperresponsiveness and abnormal peak expiratory flow following methacholine or dry-air hyperventilation challenge could be observed in adults who suffered from severe RSV infection in early childhood, as well as an increased prevalence of asthma or recurrent wheezing persisting at 18 years of age [47, 49]. Moreover, recurrent wheezing occurring early in childhood is highly predictive for lung diseases appearing in or persisting into adulthood [48].

To answer the question whether RSV might have a causal relationship with recurrent wheezing, a clinical trial on preterm infants demonstrated that children treated with palivizumab, a monoclonal antibody against RSV, exhibited reduced wheezing during their first year of life, independently of family history of atopy. However, as preterm infants are at higher risk for recurrent wheeze, it is not clear whether these findings can be applied to term infants [50]. Nevertheless, this study reveals a potential causal relationship between RSV and subsequent development of pulmonary disease. However, the immunopathological mechanisms are still not entirely understood.

### Immunopathological mechanisms involved in post-RSV lung injury

A considerable number of studies aimed at revealing the mechanisms leading to post-RSV lung disease. In a study involving children previously infected with RSV and a control group, nasopharyngeal granulocyte colony-stimulating factor (G-CSF) and IL-6 protein levels were higher in children at 1 year after severe RSV infection compared with healthy children. In addition, IL-7, IL-10, vascular endothelial growth factor and interferon (IFN)-γ levels remained high throughout the study in the post-RSV group. Although IL-13 did not differ from controls at discharge, these levels were increased 1 year after the disease [51]. The mechanisms leading to a rise in IL-13 levels remain unclear; however, altered immune responses post-RSV infection, possibly induced by airway microbiota disruption, could potentially contribute to this phenomenon. An increase in these immune mediators, especially IL-6, IL-7, IL-13 and G-CSF, are also observed in patients with asthma, which might, at least in part, explain the immunological mechanism underlying RSV-induced wheezing and asthma. However, as the study population was not exclusively infected with RSV, the exposure to different virus types might have affected the results [51]. Nevertheless, other studies on RSV revealed similar findings, where CXCL8, CCL5, IL-6 and IL-13 levels, as well as the IL-13:IFN-γ ratio were higher in infants with subsequent wheezing [52]. The role of IL-13 is further described in studies on adult mice infected with RSV A2 strain. During acute disease, IL-13 in whole lung was elevated, leading to airway hyperreactivity and mucus production [53]. The latter could be observed up to 14 days after acute disease in mouse models, along with airway obstruction persisting up to 42 days post-infection. This altered mucus production was associated with high tumour necrosis factor (TNF)-α, IL-4, IL-5, IL-9 and IL-13 levels in bronchoalveolar lavage (BAL) samples [54]. Besides leading to altered mucus production, enhanced IL-13 levels can downregulate IL-12, a highly potent antiviral cytokine [55]. An IL-13-mediated reduction of IL-12 levels might thus alter viral clearance, contributing to the persistence of RSV antigens in the lungs [53]. Other immune mediators such as IL-10 and CCL5 have been associated with post-RSV pulmonary disease. Enhanced nasopharyngeal IL-10 levels during the acute phase of RSV infection, were positively associated with post-RSV wheeze [56]. An increase in nasal CCL5 levels in children at 1 month after RSV infection was predictive of asthma diagnosis by 7 years of age [44].

A skewed immune response might also be involved in the development of RSV infection. In nasopharyngeal lavage samples from children infected with RSV, bronchiolitis was associated with an unbalanced Th1/Th2 response, described by an increase in IL-4 and a reduction in IFN-γ. Elevated levels of IL-10 and a decrease in IL-12 during acute disease further demonstrated this shift towards a Th2 response. Interestingly, these findings were independent of the risk for atopy, as children in both the control and the bronchiolitis group had one atopic parent. This deviation in the Th1:Th2 ratio during acute disease might promote the development of long-term lung disease. Therefore, severe RSV infection might induce a shift towards a Th2 immune response, which is consistent with the risk of developing asthma later in childhood [57]. In children previously hospitalised with RSV, the levels of circulating IL-4-producing T-cells were significantly more elevated compared with controls. These children also showed an increased IL-4 response to allergens and associated wheeze, contributing to the hypothesis that RSV bronchiolitis might contribute to the development of asthma or wheezing [58]. However, Castro *et al*. [59] did not describe any association between this cytokine pattern during acute infection (<12 months old) and subsequent development of asthma by 6 years of age. The authors suggest that these results might be explained by the maturation of the immune system taking place during childhood, suggesting that immune response may be influenced by age, as well as measuring techniques. Indeed, this study measured cytokine levels in peripheral blood mononuclear cells, which does not reflect lung tissue levels and might therefore not be predictive of the development of asthma.

Overall, it has been demonstrated that acute RSV infection induces a strong Th2 response with the release of cytokines such as IL-4, IL-10 and IL-13, which are associated with post-RSV wheezing, airway hyperreactivity and mucus production in both humans and mice, sharing immunological similarities with asthma. Although the causal relationship between RSV infection in early childhood and the subsequent development of asthma remains highly debated, longitudinal studies and the establishment of advanced experimental models might provide additional knowledge in the field.

### Respiratory tract microbiota and post-RSV lung disease

**FIGURE 3:**
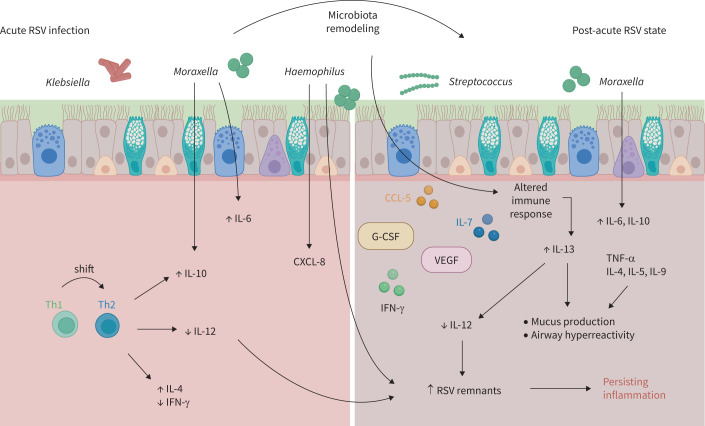
Host–bacterial interactions in post-respiratory syncytial virus (RSV) wheezing/asthma, from acute infection to chronic disease. In the acute phase of infection, the presence of *Moraxella* *catarrhalis* was linked to elevated sputum IL-10 and IL-6 levels, and *Haemophilus* was associated with increased CXCL8 levels and RSV RNA remnants [52, 60]. In addition, a shift in the Th1/Th2 response could be observed, with an increase in interleukin (IL)-4 and IL-10 levels, as well as a reduction in interferon (IFN)-γ and IL-12 nasal levels, which is also observed in asthma, illustrating the interplay between immune system and microbiota [57]. In the post-acute state, an increase in nasal interleukin and cytokine (IL-6, IL-7, IL-10, IL-13, granulocyte colony-stimulating factor (G-CSF), vascular endothelial growth factor (VEGF), IFN-γ, CCL5, CXCL8) was associated with post-RSV wheezing or asthma [51, 52]. IL-13 downregulates IL-12 levels, contributing to the persistence of RSV antigens in the lungs [53]. IL-13 also contributes to airway hyperreactivity and mucus production, the latter being additionally associated with high tumour necrosis factor (TNF)-α, IL-4, IL-5 and IL-9 levels [54]. The increased abundance in *M.* *catarrhalis* and *Streptococcus* in the post-acute state supports the notion of a remodelling of the airway microbiota after RSV infection, all in all leading to a persistent inflammatory state, with *Lactobacillus* (not shown) having a potential protective role during the acute disease [61, 62].

The important disease burden related to pulmonary sequelae after severe RSV infection and the need to understand the relationship between RSV and long-term lung damage led researchers to study the potential involvement of the respiratory tract microbiota. Airway microbiota composition during RSV infection has been correlated with post-acute lung injury [52, 60]. In a prospective study on infants with severe RSV bronchiolitis, children developing three or more episodes of wheezing by the age of 3 years harboured greater bacterial richness and diversity in sputum samples during acute disease compared with children without recurrent wheezing, independently of the use of antibiotics [52]. An increase in Proteobacteria, along with a higher abundance in *Haemophilus*, *Moraxella* *catarrhalis* and *Klebsiella* was observed in children with post-RSV wheezing, whereas the sputum of children without wheezing included more Firmicutes [52]. The microbiota profiles were associated with a pattern of inflammatory cytokines, as the relative abundance of *Haemophilus* was significantly associated with CXCL8, and *M.* *catarrhalis* with IL-6 and IL-10 (figure 3) [52].

RSV latency has been shown in the murine lung up to 100 days post-infection, which is similar to observations made following influenza virus disease [63]. This delay in RSV clearance was also observed in children at 3 weeks after hospitalisation for RSV infection, where the presence of RSV correlated with a *Haemophilus*-dominated nasopharyngeal microbiota during acute disease [60]. This finding reveals the potential role of airway microbiota in viral clearance and therefore the persistence of an inflammatory state. However, as *M.* *catarrhalis* and *Haemophilus* *influenzae* colonise airways of both healthy children and people with asthmatic, it is not clear whether this microbial pattern is specific to RSV-induced asthma [64–66].

Analysis of microbiota composition during recovery of RSV infection, provided modest insight into the microbiota dynamics following viral infection. Although this study included children infected with different virus types, including RSV, children with a higher relative abundance in nasopharyngeal *Moraxella* and *Streptococcus* 3 weeks after viral bronchiolitis had a greater risk of recurrent wheezing and asthma by age 3 years. Interestingly, the abundance of these two bacteria during the acute infection phase was not associated with subsequent lung disease [61]. This supports the notion respiratory microbiota remodelling following acute viral infection, and thereby its involvement in the pathogenesis of post-viral lung disease. On the other hand, as this study did not include samples prior to viral infection, the increased abundance in *Moraxella* and *Haemophilus* might be an indicator in children already at risk for the development of asthma. Additionally, the presence of recurrent wheezing and asthma at 3 years of age indicate a severe condition, implying that the observed microbiota alteration might be relative to disease severity.

The acute and post-RSV increase in *M.* *catarrhalis*, and most importantly the association with subsequent lung disease, suggests its role as a potential key bacterium in this condition. The relevance of *Moraxella* is further reiterated by studies investigating the clinical effect of a decrease in this bacterial genus. In children hospitalised with RSV infection, azithromycin therapy during acute disease was correlated with a reduction in *M.* *catarrhalis* abundance in the nasal microbiota and lower odds of subsequent recurrent wheezing at 1 year after infection [61, 67]. *Moraxella* is a common nasopharyngeal genus in healthy children and colonises their nasal microbiota up to the age of 3 years, along with a co-colonisation of *H.* *influenzae* [64, 65]. In addition, *Moraxella* abundance remains stable despite URT infections, and it can protect itself from antibiotics through biofilm formation [65]. *M.* *catarrhalis* might therefore act as a pathobiont, being a benign commensal bacteria in children's airways, and taking the harmful lead after viral infection.

Contrary to potential harmful associations of bacteria with post-RSV lung disease, the association with presence of *Lactobacillus* may be beneficial. In nasopharyngeal samples, an increased *Lactobacillus* abundance during acute RSV infection in their first year of life was associated with reduced risk of wheezing at age 2 years [62]. Although the mode of delivery did not bias the observed results in this study, these findings highlight a possible cofounding factor, as the mode of delivery is known to shape the newborn's nasopharyngeal microbiome. Accordingly, vaginally delivered infants harbour more *Lactobacillus* in nasal cavity than children born *via* Caesarean delivery [68]. As the latter have been shown to have a greater risk to develop asthma during childhood [69, 70], the acute dysbiosis caused by RSV might shape an adverse microenvironment for lung recovery.

In adulthood, a resilient URT respiratory microbiota following RSV infection has been described [71]. The immature immune system and early stage of lung development in infants might in fact partially explain the enhanced vulnerability for post-RSV lung injury, but also for post-viral dysbiosis and inadequate resolution of inflammation.

Taken together, a strong association exists between respiratory microbiota composition and post-RSV wheezing and/or asthma. Particularly, *M.* *catarrhalis* and *Lactobacillus* were differentially associated with pulmonary disease after RSV, playing a potential crucial role, either as a harmful or as a protective constituent of the microbiota. Moreover, *M.* *catarrhalis* was associated with an increase in IL-6 and IL-10 cytokines, both correlating with post-RSV pulmonary disease and suggesting the contribution of this bacteria to the unbalanced immune response (Th2 shift) after RSV infection (figure 3). This correlation highlights the involvement of the respiratory microbiota in post-viral-induced lung injury and supports the need to further investigate the related pathological mechanisms.

## Coronaviruses

### Coronavirus-induced lung injury

The sequential outbreak of three zoonotic respiratory viruses, SARS-CoV-1, Middle East respiratory syndrome (MERS)-CoV and SARS-CoV-2 over the last 20 years, placed coronaviruses in the spotlight of scientific research. Specifically, the associated long-term health consequences are becoming apparent and represent a significant health care issue [72–75]. Following the recent COVID-19 pandemic, millions of people worldwide suffer from a post-COVID-19 condition, also known as long COVID, which is defined as “the persistence or the development of symptoms 3 months after the acute disease” [76], including respiratory symptoms such as dyspnoea and cough, as well as multisystem symptoms involving the neurological, musculoskeletal and gastrointestinal systems [74].

Respiratory symptoms are one of the most prevalent manifestations of long COVID, and numerous studies reported persistent lung function impairment following COVID-19, the most prominent being a reduction in *D*_LCO_ [73, 77, 78] and a restrictive pattern [73]. Reduced total lung capacity, *D*_LCO_ and forced vital capacity, as well as GGOs and reticular densities in radiological imaging could be described 2–3 months after moderate-to-severe SARS-CoV-2 infection [77]. According to a recent meta-analysis, 47% of previously hospitalised COVID-19 patients suffering from long COVID have abnormal *D*_LCO_ up to 4 months after discharge [79]. Furthermore, the more severe the acute disease, the more pronounced the long-term lung function impairment, suggesting that the severity of the acute disease is associated with post-acute pulmonary function disorder [75, 79]. Indeed, persistent respiratory function impairment is more prevalent in subjects who suffered from severe COVID-19, whereas other long COVID symptoms, such as fatigue or weakness, may be present regardless of the acute disease severity [79]. Severe SARS-CoV-2 infection led to reduced *D*_LCO_ and abnormal chest radiography 12 months after the disease in 20% of subjects. Radiologically, some patients showed evolving fibrosis, along with the persistence of interstitial thickening and reticular opacity [72].

SARS-CoV-1 (SARS) and MERS, two other coronaviruses, have also been described to induce long-term lung function impairment. Following SARS and MERS infection, the main clinical feature was a reduction in *D*_LCO_, which could still be present 1 year after the disease [80–82]. Lung function impairment could even last for several years, as Zhang *et al*. [83] reported mild pulmonary function restriction (*D*_LCO_ 70% predicted value) up to 15 years after SARS-CoV-1 infection.

Lung imaging revealed pulmonary lesions after recovery from SARS infection, including pulmonary fibrosis that could be present over 1 month after hospital admission [84]. In a SARS patient cohort, 30% of the study population had airspace and reticular opacities at 6 months after the disease [85] and these abnormalities were still present in 27% of patients at 1 year following infection [81]. Zhang *et al*. [83] described that pulmonary lesions visualised on CT diminished within 1 year after SARS, before reaching a steady state. In MERS survivors, chest radiographs showed similar patterns, with lung fibrosis, GGOs and pleural thickening persisting over 1 month after the disease [86]. Overall, these findings describe a persistent morbidity after SARS and MERS infection, including long-lasting lung damage present in a significant number of patients.

### Immunopathological mechanisms in post-COVID-19 lung injury

Considering the extensive amount of post-acute COVID-19 syndrome cases worldwide, substantial effort is spent to investigate the mechanisms involved in long-term pulmonary health consequences of COVID-19 [76]. Although the exact underlying immunopathological mechanisms still remain to be clarified, current research provides first insights into the pathogenesis of long COVID.

Subjects with post-acute COVID-19 syndrome, including respiratory symptoms, have been reported to harbour lower CD8^+^ T-cell responses in peripheral blood at 4 months after mild or severe disease, which may be explained by a decreased functional capacity of the immune system, or a dysfunction in response to persistent antigen stimulation [87]. Indeed, SARS-CoV-2 RNA can persist in the lungs up to 100 days after the disease [88]. CD8^+^ T-cell response might, however, be related to the severity of the acute disease, the sample type and demographic factors such as age [89–91]. In the BAL of older individuals hospitalised for COVID-19, B-cells and CD8^+^ T-cells expressed more inflammatory molecules (such as NKG7 and granzyme K) compared with the control group at 2–3 months after infection, correlating with abnormal lung function [89]. Therefore, relative to age, the adaptive immune system might play a substantial role in the development of post-COVID condition [90]. As shown in a study on mice infected with the influenza A PR8 strain, aged murine models harboured more CD8^+^ Trm cells compared with younger animals, driving pulmonary damage [91].

Inflammatory cytokines might also be involved in the observed post-COVID respiratory function impairment. Vijayakumar *et al*. [92] analysed BAL samples of patients with persistent respiratory symptoms or abnormal CT findings in the lungs at 3–6 months after SARS-CoV-2 infection requiring hospitalisation. Respiratory dysfunction was correlated with increased numbers of activated CD8^+^ Trm cells in the BAL, potentially contributing to lung tissue damage and airway disease. In addition, airway macrophage levels, as well as T- and B-cell numbers were higher compared with controls, independently of the severity of the acute disease [92]. Markers involved in epithelial repair and fibrosis, such as matrix metalloproteinase (MMP)-3, epidermal growth factor receptor (EGFR) ligand AREG, TGFA or the epithelial marker KRT19, were upregulated in the BAL of long COVID patients. Moreover, cytokines indicating epithelial damage and cell death (lactate dehydrogenase, albumin, EPCAM and CASP3, as well as CXCL9, CXCL10, CXCL11 and IL-7) were also higher in this study population compared with the control group, suggesting ongoing respiratory disease [92].

In subjects who developed long COVID syndrome, including dyspnoea, circulating IFN-β and IFN-λ1 remained elevated 8 months after the acute disease, which was different from patients infected with other coronaviruses. At that same time point, IFN-β, PTX3, IFN-γ, IFN-λ2/3 and IL-6 were highly associated with long COVID development, indicating a persistent activation of acute inflammatory markers. As IFN-γ is associated with aberrant lung repair, this result may explain the persistence of respiratory symptoms. Additionally, CD8^+^ T-cells were activated up to 8 months after infection in long COVID patients, which might be explained by a persistent immune activation secondary to underlying inflammation [93]. As shown in a study on nonhospitalised COVID-19 patients, 5% of the recovered individuals still had a positive SARS-CoV-2 PCR test at 90 days post-disease, along with an increase in SARS-CoV-2 CD8^+^ T-cell responses compared with antigen-negative subjects [94]. Schultheiß *et al*. [95] described a cytokine triad associated with post-COVID syndrome. In blood samples of patients with prior COVID-19, IL-1β, IL-6 and TNF correlated with persistent symptoms, including respiratory impairment at 8 months after the acute disease, expressing an unresolved inflammatory state. In post-COVID-19 patients with persistent respiratory symptoms, blood IL-6 levels and SARS-CoV-2-specific CD8^+^ T-cells were elevated compared with subjects with resolved COVID-19, along with elevated Ki-67 expression in CD4^+^ and CD8^+^ T-cells, and thus indicating proliferation of these cells. Moreover, this pattern was associated with decreased lung function [96].

Altogether, it has been suggested that subjects with persistent respiratory symptoms after COVID-19 harbour pro-inflammatory cytokines, increased CD8^+^ T-cell responses, as well as elevated epithelial repair and fibrosis markers. Further studies are therefore needed to define the specific immunological characteristics of post-acute COVID-19. Specifically, research on the human microbiota might add another piece to the intricate puzzle of post-COVID-19 respiratory impairment.

### Post-COVID-19 and the microbiota

**FIGURE 4:**
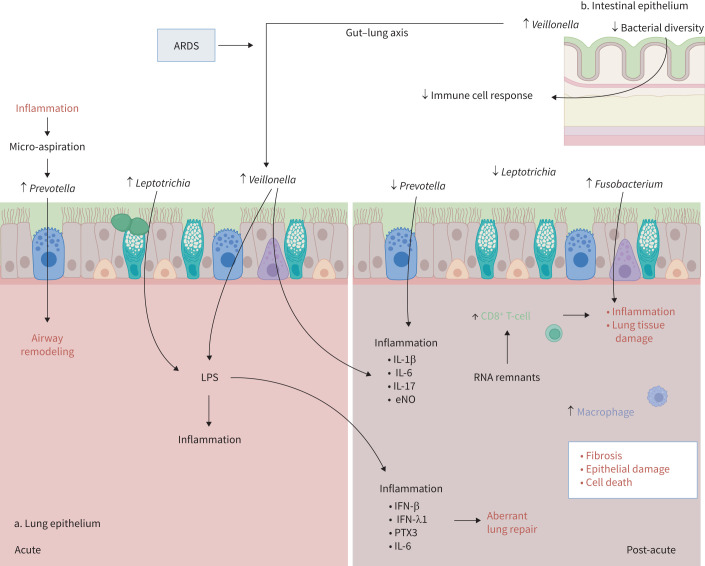
Host–bacterial interactions and post-COVID lung sequelae. During acute disease, increased abundance in *Veillonella*, *Prevotella* and *Leptotrichia* was associated with persistent respiratory symptoms. *Veillonella* and *Leptotrichia* produce lipopolysaccharide (LPS) with pro-inflammatory effects, contributing to chronic inflammation [98]. The enrichment in *Veillonella* might additionally be explained by a translocation of *Veillonella* into the lungs through disruption of the gut–lung axis [25, 28]. The overrepresentation of *Prevotella* might be driven by viral-induced inflammation and subsequent micro-aspiration of this bacterial species, leading to undesired lung tissue remodelling [11, 98, 99]. In the post-acute phase, an increase in activated CD8^+^ T-cells contributes to inflammation and lung tissue damage [93]. The persistence of SARS-CoV-2 RNA remnants may additionally activate the immune system [88]. Cytokines involved in epithelial repair and fibrosis (matrix metalloproteinase-3, epidermal growth factor receptor ligand AREG, TGFA and KRT19) are upregulated, along with markers related to epithelial damage and cell death (lactate dehydrogenase, albumin, EPCAM, CASP3, CXCL9, CXCL10, CXCL11 and interleukin (IL)-7) [92]. Inflammatory molecules, such as IL-1β, interferon (IFN)-β, IFN-λ1, IFN-γ and IL-6 are also increased in long COVID syndrome [93]. This persistent inflammatory state may be related to the decrease in *Prevotella* [11]. The increase in *Fusobacterium* might further contribute to inflammation and lung tissue damage [100]. Finally, decreased bacterial diversity in the gut might impair immune cell activation in the lungs through the gut–lung axis [101]. ARDS: acute respiratory distress syndrome; eNO: exhaled nitric oxide.

Until now, only a few studies have investigated the respiratory microbiota composition post-acute SARS-CoV-2 infection. Yet such findings may provide useful insights into the role of the microbiota in post-COVID-19 lung injury, persistence of sequelae and recovery. A decreased bacterial diversity was observed at 3 weeks after the disease in the URT of previous mild-to-moderate COVID-19 patients when compared with controls [97]. Haran *et al*. [98] observed that increased abundance in oral *Veillonella*, *Prevotella* and *Leptotrichia* during acute COVID-19 disease in hospitalised subjects was associated with persistent respiratory symptoms at 10 weeks after the disease. The ability of *Veillonella* and *Leptotrichia* to produce lipopolysaccharide (LPS), which has pro-inflammatory effects, might contribute to post-acute pulmonary impairment through chronic inflammation (figure 4). Analysis of the oropharyngeal microbiota in recovered COVID-19 patients with prior mild or moderate disease revealed that *Prevotella* dominated during the acute state of the disease and in healthy controls, whereas *Fusobacterium* was increased and *Leptotrichia* decreased in the recovered subjects [102]. Similarly, oral microbiota composition at 1 year after COVID-19 was increased in *Fusobacterium*, a known butyrate producer, and a decrease was observed in *Prevotella* in recovered patients compared with healthy controls [103]. As a commensal of the human oral microbiota, *Fusobacterium* may have a protective function against invading pathogens, and can regulate the immune system by producing butyric acid. However, *Fusobacterium* subtypes have been shown to contribute to disease development and inflammation [100]. As the findings from the above study did not specify the *Fusobacterium* subtypes, it might therefore reflect an altered microbiota, with an increase in potentially pathogenic *Fusobacterium*.

*Prevotella* is a commensal airway bacteria colonising healthy individuals [99]. After lung transplantation, a shift away from *Prevotella*-dominant airway microbiota is associated with lung inflammation, whereas a *Prevotella*-dominant phenotype was characterised by airway remodelling [11]. Hence, the observed decrease in URT *Prevotella* after COVID-19 may be related to a persistent inflammatory state in the airways. Moreover, the presence of *Prevotella* in the LRT of healthy subjects has been associated with a subclinical pro-inflammatory state, with increased cytokine levels relevant for Th17 differentiation, including IL-1β, IL-6 and IL-17, as well as the fraction of exhaled nitric oxide, a marker of Th2 lung inflammation [104, 105]. Different sample types (for example URT *versus* LRT) may potentially explain these discrepancies. Furthermore, attention should be paid to population studies do understand how the lung microenvironment differs between health and disease. As the altered microenvironment in disease might affect the microbiota differently than in healthy individuals, comparing health and disease groups in a specific study population may lead to divergent results.

The cytokine triad (IL-1β, IL-6 and TNF) reported by Schultheiß *et al*. [95] in post-COVID-19 subjects may therefore be associated with *Prevotella*, demonstrating the role of specific bacterial species in this condition. On the other hand, the presence of *Prevotella* and the associated direction of causality remains unclear, as micro-aspiration of oral *Prevotella* may also cause airway inflammation [99]. Accordingly, the increase in *Prevotella* observed by Haran *et al*. [98] during acute disease might be driven by SARS-CoV-2-induced inflammation, subsequently leading to an overrepresentation of this bacterial species and ultimately contribute to lung tissue remodelling with subsequent persistent post-COVID-19 respiratory impairment [11].

The involvement of the gut microbiome in lung health might additionally explain the discussed findings (figure 4). In fact, several studies have characterised the gut microbiota and its association with lung function impairment and persistent symptoms after COVID-19. In previously hospitalised COVID-19 patients, gut microbial diversity was reduced in patients with persistent respiratory symptoms at 3 months after the disease, along with a reduction in butyrate-producing species [101]. Vestad *et al*. [106] described an enrichment in *Veillonella* species in the gut of subjects with abnormal *D*_LCO_ at 3 months post-infection. In relation to the gut–lung axis and the disruption of gut permeability observed in severe lung infection and ARDS, the impairment in *D*_LCO_ might well be explained by a translocation of *Veillonella* species into the lungs [28]. These bacteria are known to enhance LPS levels and induce IL-6 production with subsequent lung injury [28, 98]. The bacterial shift between these two microbial environments might thus explain the persistent activated immune response described above.

The airway and the gut microbiota might also be implicated in the CD8^+^ T-cell responsiveness described above [107, 108]. In fact, germ-free mice demonstrated a poorer CD8^+^ T-cell recall response and less IFN-γ production, and the administration of short-chain fatty acids improved CD8^+^ T-cell memory response [107, 108]. Furthermore, disturbed gut microbiota caused an altered ability to regulate immune cell function, especially natural killer cells and CD8^+^ T-cells, in the lung cancer setting [109]. The gut–lung axis is therefore an important player in determining respiratory immune cell function. Finally, as shown by Kusakabe *et al.* [110], fungal micro-organisms in the gut might also be implicated in the immunological response after severe COVID-19, thus possibly contributing to long-term pulmonary sequelae.

Interestingly, the lack of any study on the human microbiota during or after SARS or MERS reflects the knowledge gap on the involvement of the respiratory microbiota in long-term pulmonary sequelae. Given the similarities among viruses belonging to the coronavirus family, we speculate that SARS-CoV-1 and MERS-CoV may have comparable immunopathological mechanisms with similar impacts on the microbiota as discussed above [111]. The outbreaks of three novel coronaviruses in the last 20 years and their subsequent morbidity must raise our awareness of the need to understand the mechanisms leading to long-term lung injury after coronavirus infection. The respiratory microbiota, with its immunoregulatory ability, can influence lung recovery and should therefore be taken into consideration as a key factor in future research.

Taken together, post-COVID-19 patients have a distinct airway microbiota compared with controls, which could be associated with persistent respiratory symptoms. Increased abundance in LPS-producing bacteria during acute disease might participate in the persistence of lung inflammation, and the decrease in *Prevotella* could reflect a pro-inflammatory state in the lungs. Last, the gut–lung axis has been shown to contribute to the observed immunopathological and clinical findings, especially in subjects who suffered from severe disease. This supports the hypothesis that the microbiota plays an important role in pulmonary recovery after COVID-19.

## Future perspectives and key questions in the field

### Airway microbiota and post-viral lung diseases: cause or consequence?

This article highlights the significance of host–bacterial interactions in the development of lung sequelae after viral infections, reiterating the importance of the bacterial microenvironment as a fundamental cofactor in altered immune responses contributing to post-viral lung injury. Most research has focused on microbiota analysis during acute disease, pointing out the lack of additional studies on airway microbiota dynamics after viral infections. Whether the virus induces altered microbiota with subsequent inappropriate immune response, or the microbiota itself represents a predisposition for viral infection is highly debated. Similarly, subjects with chronic lung disease, as well as the elderly population, are at higher risk to develop severe respiratory viral infection and post-viral lung disease. These conditions might be linked to a disbalanced microbiota, suggesting a possible predisposition to post-viral lung disease, as shown in a study demonstrating an enrichment of Firmicutes in the lower airways of COPD subjects compared with healthy controls [112]. Longitudinal studies aimed at answering these questions may provide additional data on temporal microbiota dynamics and the direction of causality in post-viral lung diseases.

### Airway microbiota research in post-viral lung diseases: current challenges and future perspectives

The current knowledge on mechanisms involved in post-viral lung injury is frequently based on murine models. The lack of studies on clinical samples indicates the need for further investigation on the immunopathological mechanisms in previously infected patients. Given the complexity of host–bacteria interactions, exploration of the human respiratory microbiota remains a challenging research area. In fact, airway microbiota is susceptible to inter- and intra-individual changes, limiting the characterisation of a ubiquitous normal microbial pattern [113]. As observed during childhood, environmental factors such as familial environment and seasons alter bacterial colonisation [114]. Studies using human models of viral challenge are critical in our understanding of the discussed underlying mechanisms of post-viral lung diseases as they provide a well-controlled study set-up and may enhance our knowledge on the causal relationship between virus infection and long-term alterations of the respiratory tract microbiota. In a rhinovirus challenge, Molyneaux *et al*. [115] demonstrated a rise in bacterial burden and an outgrowth of *H.* *influenzae* in COPD subjects, whereas this was not observed in healthy controls, suggesting that rhinovirus infection modulates the lung microbiota in COPD patients. Another rhinovirus challenge on healthy individuals did not show any impact of rhinovirus infection on the nasal microbiota, highlighting the need of further studies to understand the effect of chronic lung diseases in response to viral infection [116]. Interestingly, although immune status and in particular neutrophil activation, determined the severity of RSV infection in a human challenge model, the pre-infection airway microbiota was not associated with altered susceptibility to RSV infection [117].

Additionally, study participants might be affected by extrinsic factors such as diet or the use of antibiotics and other medications. This might explain the observed discrepancies between study groups. In fact, the use of corticosteroids as a common treatment for viral lung diseases might influence the microbiota. In a study using human and mice COPD models, inhaled corticosteroids (ICSs) drove the expansion of *Streptococcus* in the lower airways [118]. Results from a randomised trial on subjects with COPD demonstrated that ICS use was linked to a reduced bacterial diversity and greater microbiota shifts (increase in Firmicutes and reduction of Proteobacteria) compared with subjects receiving only formoterol [119]. Moreover, in subjects with asthma, the fungal but not the bacterial community composition changed significantly after ICS use [120]. Together with the lack of standard sampling and analysis procedures [121], these potential biases might restrict the comparison of results from distinct cohorts. The establishment of consortia and study groups could promote the implementation of standardised research methods, supporting microbiota research in post-viral lung diseases.

The main respiratory viruses of concern seem to have distinct effects on lung health, immune responses and the microbiota. This review primarily focuses on SARS-CoV-2, RSV and influenza virus, the last two being among the most common causes of respiratory infections worldwide [122, 123]. Nevertheless, other aetiologies of airway infection, such as rhinovirus and bacterial infection might as well affect the microbiota and have an effect on post-viral lung diseases [123]. However, given the paucity of data we have not included this in the current literature review. Additionally, viral variants [124], as largely observed in SARS-CoV-2, may induce different immune responses, increasing the complexity of research on post-viral lung injury. However, current progress in diagnostic microbiology allows precise characterisation of viral strains, improving the accuracy of immunological and microbiome research.

Furthermore, the respiratory microenvironment not only comprises bacteria, but also viruses and fungi [125, 126]. To date, respiratory microbiota research primarily focuses on bacterial components, neglecting other airway and gut commensals, such as fungi and viruses, as well as inter-kingdom interactions which have been shown to play a substantial role in lung disease [110, 127–130]. Further research is therefore needed to assess the interplay between commensal bacteria, fungi and viruses and the effect on respiratory health, specifically in post-viral lung diseases.

### Post-viral lung diseases: the microbiota as a potential therapeutic target

We underscored the need to consider post-viral lung diseases not as a straightforward consequence of altered immune responses, but as an outcome involving a complex network of clinical presentation, immunology and microbiology. By understanding these intricate connections, it seems conceivable to think of the airway microenvironment as a potential target for preventive, predictive and therapeutic strategies in post-viral lung diseases. Although limited, recent studies have demonstrated the potential for therapeutic approaches targeting the microbiota in chronic lung diseases through the use of prebiotics or faecal microbiota transplantation [131]. These promising results support the need for further studies investigating this emerging field in the treatment and prevention of post-viral pulmonary diseases.
